# Opinion: Standardizing the definition of gene drive

**DOI:** 10.1073/pnas.2020417117

**Published:** 2020-11-18

**Authors:** Luke S. Alphey, Andrea Crisanti, Filippo (Fil) Randazzo, Omar S. Akbari

**Affiliations:** ^a^Arthropod Genetics Group, The Pirbright Institute, Woking GU24 0NF, United Kingdom;; ^b^Department of Life Sciences, Imperial College London, London SW7 2BU, United Kingdom;; ^c^Department of Molecular Medicine, University of Padova, 35122 Padova, Italy;; ^d^Leverage Science, LLC, Berkeley, CA 94705;; ^e^Division of Biological Sciences, Section of Cell and Developmental Biology, University of California San Diego, La Jolla, CA 92093

Gene drive has become a hot topic in the popular press and the scientific literature, yet little consensus vocabulary on the subject exists. As members of the gene drive community, we have developed a core set of definitions to help stakeholders discuss the topic and communicate using a common understanding of terms. A standard consensus definition of gene drive and a glossary of terms, noted here, will be of great practical use to a field that has implications for both researchers and the general public. If we don’t clarify these terms, we risk hampering the field, confusing the public, and possibly losing a technology that may help solve some of the world’s most intractable problems in public health, conservation, and food security.

**Figure:**
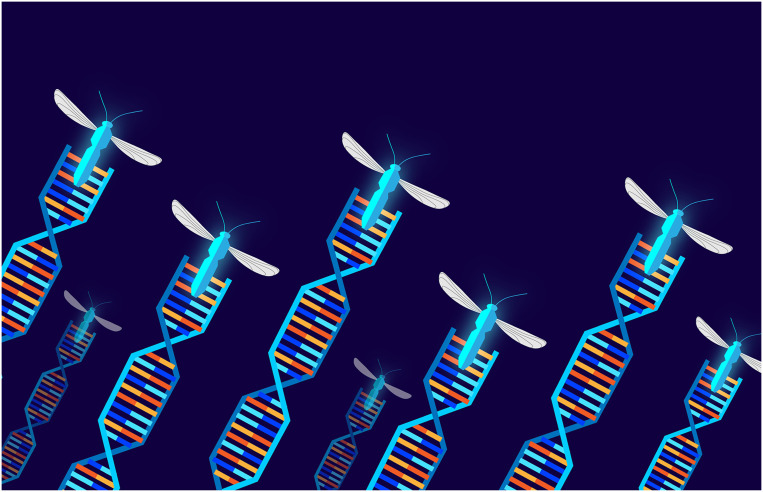
We need to clarify gene drive terms, or we risk hampering the field, confusing the public, and losing a technology that may help solve otherwise intractable problems in public health, conservation, and food security. Image credit: Stephanie Gamez (University of California San Diego, La Jolla, CA).

Loosely, gene drive refers to a phenomenon whereby a particular heritable element biases inheritance in its favor, resulting in the gene becoming more prevalent in the population over successive generations. Thus, the gene is being “driven” to progressively increase its frequency in the population. Biasing inheritance may involve, for example, more than the familiar Mendelian 50:50 inheritance chance or reducing the fitness of alternative genotypes without directly distorting Mendelian inheritance. Highly efficient gene drives can bias inheritance so heavily in their favor that the heritable element can rapidly reach high frequency, close to doubling its frequency from one generation to the next. The speed of this process is inversely correlated with generation time of the organism (for example, mosquitos have a generation time of 2–4 weeks and rats have a generation time of 12 weeks, whereas some whale species can have generation times of 50 years or more). However, in all cases, acquisition of the heritable element is expected to occur much faster than with more conventional types of genetic change driven by natural selection.

Without a common understanding of standard terms, policy discussions on gene drive can become difficult and confusing.

The general repertoire of allelic frequencies in a given population usually does not change rapidly over generations. Changes in allelic frequency can and do occur without gene drive, however, through random chance (“genetic drift”) or selection. The presence of either a positive or a negative selection pressure acting on a given allele can increase or decrease frequency, respectively, but such changes are normally relatively slow. Notably, both natural and engineered gene drives can spread genes that have an adverse effect on population fitness to the point of causing its collapse.

Many types of gene drives exist in nature, for example transposons, sex distorters, toxin-antidote systems, and homing nucleases (Table 1 and Dataset S1). These have attracted interest for decades from both fundamental and applied research perspectives. In addition to basic biological studies examining their role in evolutionary processes, the drive molecular mechanisms could be exploited to develop applications that have a range of potential benefits for health and the environment. Applications directed toward control of mosquito-borne diseases have been a particular focus of gene drive research.

**Table 1.** Characteristics and examples of engineered population modification/suppression technologies

Approach	Examples	Temporal dynamics	Geographic reach
Gene drives	HEG#, Medea, CleaveR	Low-threshold	Nonlocalized
	Translocations, Underdominance#, UD^MEL^	High-threshold	Localized
	Daisy#, split-drive#, killer rescue	Self-limiting	
Nondrives	SIT#, RIDL#, fsRIDL#, pgSIT#		

Two broad types of engineered approaches exist to modify/suppress populations—one requires gene drive, and the other relies on nondrive technologies. Multiple examples of these types of systems exist, which work over different timeframes, including: Low-threshold (predicted to spread from a small release), to high-threshold (predicted to spread into a population only when the transgene is present above a critical threshold), to self-limiting, which can only spread or persist in population for a short period. These systems can fall under two broad categories, from nonlocalized (predicted to spread beyond boundaries) to localized (predicted to spread within a local population). Some gene drives (and other genetic methods) can be used for population suppression, at least in some forms (indicated by #). For more details on the various examples and terminology see Dataset S1.

The advent of CRISPR/Cas9 technology provides a powerful mechanism for harnessing gene drive by providing unprecedented flexibility to target user defined locations of a target genome and at the same time expediting the development of effective solutions. The technology is new, difficult for nonspecialists to conceptualize given the variety of possible genetic mechanisms being explored, and can potentially interfere with the evolutionary trajectory of animal species. The research community, together with policymakers and funders, have provided guidance documents that discuss issues such as the phased testing process, benefit and risk assessment, regulatory requirements, ethics, stakeholder engagement, and governance (1–13). However, the lack of a common definition poses a practical dilemma to researchers, policymakers, and other stakeholders.

The National Academy of Sciences report on gene drives (2) noted:

“In reviewing the history of research on what are now called selfish genetic elements, the committee noted differences in the use of terminology and definitions. *Drive*, *gene drive*, *meiotic drive*, *driving Y chromosome*, *selfish gene*, *selfish genetic elements*, and related concepts often have overlapping definitions depending on the historical period and the scientific context in which the terms are used.”

Furthermore, 11 published policy guidance documents generated by various national and international organizations that address gene drive do not use a common definition (1–11). The translation of gene drive terms into other languages poses additional problems as researchers use different analogies to describe the same technology (14). Without a common understanding of standard terms, policy discussions on gene drive can become difficult and confusing. Discussions of policy become critical as governments begin to regulate the technology and as multilateral treaty organizations begin to consider whether and how to use the technology.

The scientific community itself has abetted this confusion. As the literature in the gene drive field has grown rapidly, new terms associated with gene drive have proliferated, scattered across the literature. At this critical early stage in development of gene drive technologies, researchers need a consistent and common language to engender trust with the public.

## Reaching Consensus

These types of challenges are not new to science. For example, the malaria and immunology fields have recognized and addressed similar challenges in the past (15–17). For instance, the World Health Organization (WHO) Global Malaria Program produced a World Malaria Terminology glossary to prevent confusion in the field given that “medical language must be adaptable so that it can keep pace with the constant increase of our knowledge and with the continual revision and evolution of our concepts” (15). The immunology field standardized its nomenclature of monoclonal antibodies and cluster of differentiation antigens, respectively, because independent laboratories were giving different names for the same entity (16, 17).

To address this challenge in the gene drive community, we reached out to more than 60 researchers and stakeholders on preferred definitions for gene drive and created a glossary of terms. The following definitions have been agreed upon by these signatories and are proposed for widespread use. (Because the field continues to evolve, we have composed a living document for signatories that will be updated periodically.*) For each usage, we include a technical definition as well as a less technical version that may be more accessible to a nonscientific audience.

“Gene drive” is used both to describe a process or phenomenon (the biological activity of gene drive) and to describe an object (“a gene drive”). The term sometimes is also used to describe a management tool or intent for product development or regulatory purposes.

I. **Process or Phenomenon:** A gene drive is a phenomenon of biased inheritance in which the prevalence of a genetic element (natural or synthetic) or specific alternate form of a gene (allele) is increased, even in the presence of some fitness cost. This leads to the preferential increase of a specific genotype that may determine a specific phenotype from one generation to the next and potentially spread throughout a population. In other words, *a gene drive is a process that promotes or favors the biased inheritance of certain genes from generation to generation.*
II. **Material Object:** A gene drive is composed of one or more genetic elements that can cause the process of biased inheritance in its favor. The set of necessary elements may be referred to as a gene drive system or simply a “gene drive.” Note that the presence of gene drive elements will not necessarily cause gene drive—many gene drive systems will cause the gene drive phenomenon only under specific circumstances (e.g., if they are present in the population above a certain threshold frequency, or if fitness costs are below a certain threshold, or if all drive componets are present in the organism). Note also that gene drive, when defined as an object, need not always confer preferential transmission. Gene drives must ensure biased inheritance under at least some circumstances but not necessarily all circumstances. For example, some gene drive systems confer preferential inheritance only when present in the population above a threshold frequency. That is, *a gene drive is any genetic element able to bias its inheritance within a population.*
III. **Intention:** A gene drive may be intended as a management tool to achieve a particular goal. A gene drive may include additional “cargo” elements, in addition to the drive components, that are intended to introduce new trait(s) into an interbreeding population so as to effect a change in the characteristics of the population. A gene drive also may cause effects directly, for example by inserting into and disrupting a target gene. Thus, *a gene drive is a tool to effect certain changes in a population.*

## Important Caveats

Additional concepts can help further improve the understanding of gene drives. Again, gene drives are a natural phenomenon. They have arisen through entirely natural processes of mutation and selection; indeed many types exist (transposons, toxin-antidote systems, homing nuclease, and sex distorters), and gene drives and relics of old gene drives are widespread in nature. Naturally occurring gene drives can induce the development of potent suppression systems in response to the selection pressures they impose on the population. Modern molecular biology now allows researchers to mimic various types of natural gene drives in the laboratory. A gene drive system that is created through recombinant DNA techniques is called an “engineered” or “synthetic” gene drive.

A number of basic criteria foster efficient synthetic gene drive. First, the organism must have an inheritance pattern that can be biased; this typically means that it can reproduce sexually. Many plants and some animals that use other means to reproduce cannot be altered in this way. Second, to be practical, the organism must have a short generation time**.** Gene drive can lead to an increase in the frequency of a specific genetic element from one generation to the next, but speed depends on the generation time of the organism. Consequently, discussions of gene drive applications have focused on species such as tropical/subtropical mosquitoes, with generation times around two weeks to a month, and so multi-generation effects are potentially visible in a few years, rather than organisms for which a similar number of generations might take decades or even centuries.

Gene drive is not a monolithic phenomenon. Just as many forms of gene drive exist in nature, many types of engineered gene drive have been conceived. Gene drives can be subclassified by biologic mechanism, molecular configuration, intended use, and time of action—pregametic versus postgametic. Other classification schemes are possible.

Gene drive is not synonymous with either persistence or spread of a trait through a population. Persistence is the ability of a genotype or phenotype to continue over multiple generations. Spread refers to the ongoing movement of the genotype through increasingly larger segments of the population. Thus, there is a relationship between persistence and spread. Both persistence and spread are possible without gene drive, through natural selection for example. Some gene drive systems have the potential to persist indefinitely. Other forms are not intended or able to persist indefinitely, even in the absence of heritable resistance or genetic change (mutation). Some gene drive systems have the potential to reach fixation in a population (fixation means that no other gene variants of the gene drive are present), whereas researchers predict that others persist at less than 100% frequency. Not all forms of gene drive have the potential to spread widely; high fitness costs, for example, may prevent spread despite strongly biased inheritance. Gene drive systems that have limited persistence also are expected to have limited ability to spread within the environment.

Given that the terms associated with the gene drive field are scattered through the literature, we have compiled a glossary of 78 terms along with their definitions (Dataset S1). This list includes terms that are specialized for the field as well as more standard genetic terms that are foundational to discussion around gene drive concepts. This list is not meant to be exhaustive but rather is intended as a general reference for stakeholders.

Outlining these definitions in detail will be an ongoing process as this area of research continues to develop, and this is only a start. However, achieving broad agreement on the use of basic terminology is fundamental to communicating effectively about this new technology. Only by doing so can we help this important and potentially impactful field progress.

## Supplementary Material

Supplementary File

## Footnotes

*To address the identified need for consistent terminology in the gene drive field, interested parties are encouraged to become signatories to these definitions by visiting https://www.geneconvenevi.org/articles/gene-drive-nomenclature/.

## Change History

December 8, 2020: The license has been updated.
